# Gene body demethylation increases expression and is associated with self-pruning during grape genome duplication

**DOI:** 10.1038/s41438-020-0303-7

**Published:** 2020-06-01

**Authors:** Luming Zou, Wenwen Liu, Zhan Zhang, Everard J. Edwards, Elias Kirabi Gathunga, Peige Fan, Wei Duan, Shaohua Li, Zhenchang Liang

**Affiliations:** 1 Beijing Key Laboratory of Grape Science and Enology and CAS Key Laboratory of Plant Resources, Institute of Botany, Chinese Academy of Sciences, Beijing, 100093 PR China; 2 University of the Chinese Academy of Sciences, Beijing, 100049 PR China; 3 College of Life Science, Shanxi Normal University, Shanxi, 041004 PR China; 4 CSIRO Agriculture & Food, Locked Bag 2, Glen Osmond, SA 5064 Australia; 5 Sino-Africa Joint Research Center, Chinese Academy of Sciences, Wuhan, 430074 PR China

**Keywords:** DNA methylation, DNA methylation

## Abstract

A colchicine-induced autotetraploid grapevine exhibiting potentially valuable agronomic traits for grape production and breeding, including self-pruning, was identified. This study investigated DNA methylation variation and its role in gene expression during self-pruning in the autotetraploid grapevine. We used RNA-Seq to estimate differentially expressed genes between diploid and autotetraploid grapevine shoot tips. The genes showing increases in the autotetraploid were mainly related to stress response pathways, whereas those showing decreases in the autotetraploid were related to biological metabolism and biosynthesis. Whole-genome bisulfite sequencing was performed to produce single-base methylomes for the diploid and autotetraploid grapevines. Comparison between the methylomes revealed that they were conserved in CG and CHG contexts. In the autotetraploid grapevine, hypodifferentially methylated regions (DMRs) and hyper-DMRs in the gene body increased or decreased gene expression, respectively. Our results indicated that a hypo-DMR in the *ACO1* gene body increased its expression and might promote self-pruning. This study reports that hypo-DMRs in the gene body increase gene expression in plants and reveals the mechanism underlying the changes in the modifications affecting gene expression during genome duplication. Overall, our results provide valuable information for understanding the relationships between DNA methylation, gene expression, and autotetraploid breeding in grape.

## Introduction

Polyploidy or whole-genome duplication (WGD) has been proposed to be one of the primary forces in plant evolution^1^. A previous study suggested that more than one WGD event occurred prior to the diversification of angiosperms and that this phenomenon contributed to the current dominance of seed plants^2^. This process of genome duplication that occurred at least several million years ago is referred to as paleopolyploidization. Most paleopolyploid plants underwent a subsequent process of diploidization, during which they lost their polyploidy, and secondary diploid-like genome was restored. This process results in the loss of some duplicated genes^3^. Following WGD, various processes can affect gene function and transcription. For example, the shuffling of regulatory sequences can change the expression patterns of duplicated genes, leading to sub-functionalization, and the shuffling of the functional domain sequence in a duplicated gene can change its function, leading to neo-functionalization^4^. However, dosage-sensitive genes, such as the circadian clock genes of *Brassica rapa*^5^, and transcription factors^6^ are retained during dipolyploidization.

Neo- (or recent) polyploids maintain two or more parental genomes and can be divided into two main categories: autotetraploids and allopolyploids. Autopolyploids contain more than one genome from the same species, originating from an intraspecies WGD event^7^. The interspecies hybridization of autotetraploids generates allopolyploids. Polyploidy presents advantages in allowing changes in gene function, thus avoiding limitations on evolutionary selection due to the presence of multiple gene copies^8^. Polyploidy can also be introduced artificially, and this process is increasingly being employed in agricultural production and breeding^9^.

There are many methods that can be used to induce artificial polyploids^10–12^. Colchicine induction is one of the most effectively used methods for obtaining polyploids^9^. Artificial polyploids typically exhibit greater variation in morphology than diploids and can be used in industrial applications or as valuable breeding material. In some cases, polyploidy increases the synthesis of metabolites that benefit humans. For example, the production of podophyllotoxin^12^, tropane alkaloids^13^, and methoxylated flavones^14^ is increased in tetraploid *Linaceae*, *Hyoscyamus niger*, and *Labiateae*, respectively.

In addition, the plant organs of polyploids are often enlarged, which can also confer agronomic benefits. Fruit size^15^, the number of spikelets^16^, and leaf area^17^ are increased in autotetraploid mulberry, *Paspalum plicatulum*, and birch, respectively. In our laboratory, we have found many morphological variations in colchicine-induced autotetraploid grapevine.

In perennial horticulture, pruning is often essential to maintain production, ensure vegetative/reproductive growth balance, and maximize fruit quality. However, pruning is associated with a high labor cost. The abscission of plant organs such as leaves, fruits, and flowers occurs naturally if they are mature or unnecessary for the plant. Conversely, self-pruning is a type of abscission that occurs at the shoot tip. Self-pruning has been observed in citrus, chestnut, and tomato^18–20^. Self-pruning is also found in grapevines, as described in this study, and can increase horticultural production efficiency if utilized effectively to reduce manual pruning.

As generally observed in the abscission of plant organs, an abscission zone (AZ) is required for self-pruning to occur^18^. The plant hormone balance plays an important role in plant organ abscission. Ethylene triggers AZ formation, whereas auxin suppresses abscission in many plants^21,22^. A decline in auxin increases the ethylene response and subsequently promotes AZ differentiation^23^. During abscission, ethylene increases the expression of an ACC oxidase (*ACO*) gene and the activity of *ACO* while inhibiting the transportation of auxin^24^. In the current study, the expression of the *ACO1* gene was found to increase during self-pruning in the autotetraploid grapevine shoot tip.

DNA methylation plays an important role in the development of a plant phenotype^25^. Gene-associated DNA methylation can occur in the promoter and/or within the transcribed gene body (including both introns and exons). Increasing DNA methylation in the promoter usually decreases gene expression by inhibiting transcription factors and binding repressors^26^. In addition to the effect of the promoter methylation state, recent studies have revealed that gene expression levels are positively correlated with the gene body methylation status in human cell lines^27^.

Genomic structural and epigenetic changes have commonly been observed in newly induced polyploids^3^. However, few studies have revealed the relationship between epigenetic changes and gene expression variation in artificial polyploids^28^.

In the present study, a colchicine-induced autotetraploid grapevine was found to show reduced lateral shoot growth and a self-pruning morphology. We detected DNA methylation variation by whole-genome bisulfite sequencing (WGBS) and investigated its role in gene expression modification and self-pruning. We found that differentially methylated regions (DMRs) in the gene body were correlated with changes in gene expression. For example, a hypo-DMR in the gene body of *ACO1* resulted in a fourfold increase in expression. This upregulation of *ACO1* might increase the release of ethylene and lead to self-pruning in this autotetraploid grapevine. We also report that DMRs in the gene body were negatively correlated with gene expression. These observations provide valuable information revealing the relationship between gene body DNA methylation variation and gene expression. Self-pruning in this autotetraploid grapevine is expected to contribute to grape breeding and production in the future.

## Results

### Morphological differences between the diploid and the autotetraploid

**Fig. 1:**
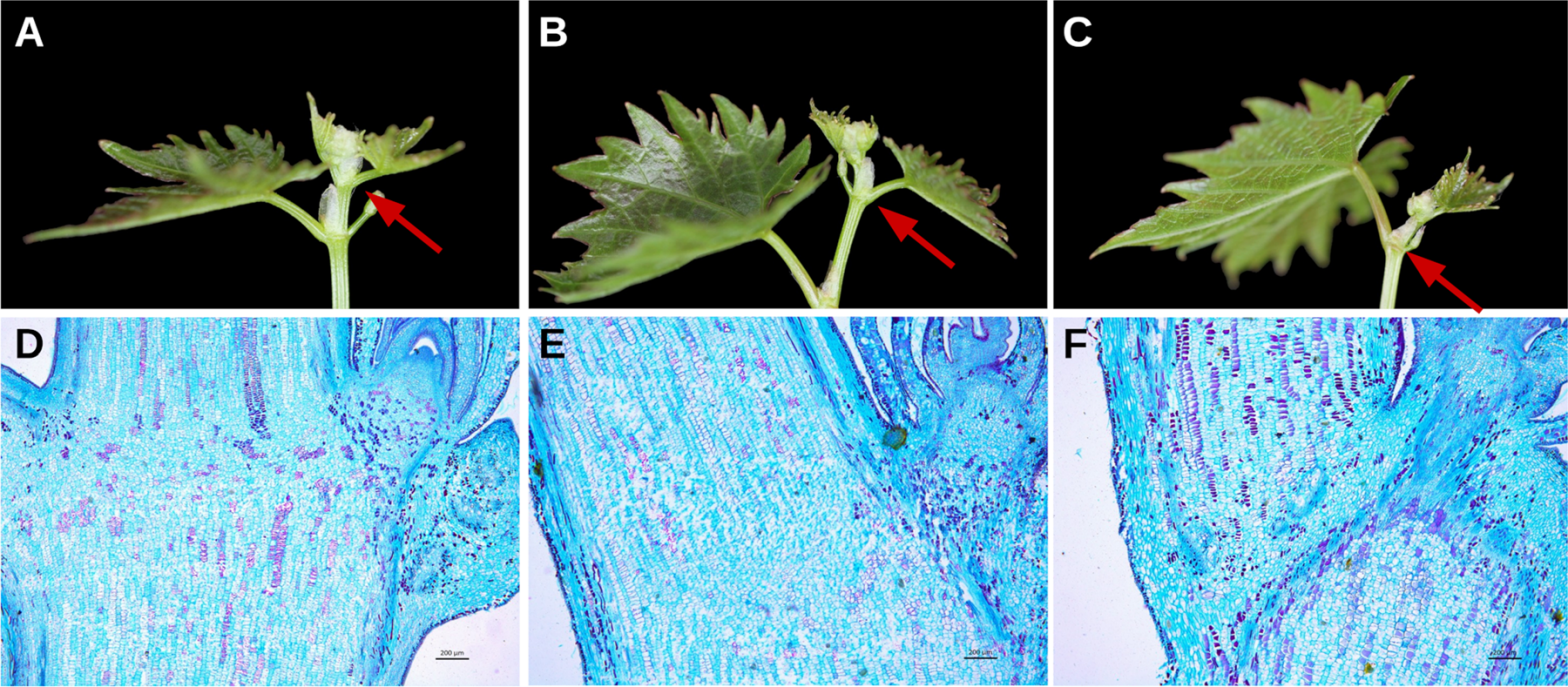
Tips of ‘Jingyanjing’ and its autotetraploid. Normal growing tips of (**a**) ‘Jingyanjing’ (2×), its (**b**) autotetraploid (4×), and creased growth tip of (**c**) 4×. Red arrows point out the parts shown in their longitudinal sections in the row below. The anatomical structures of (**d**) 2× and (**e**) 4× were not significantly different. **f** Abscission zone clearly appeared in the section of the 4× tip with ceased growth

‘Jingyanjing’ (2×) grapevine is a typical *Vitis vinifera* diploid accession. Considerable differences in shoot growth were observed between 2× and the autotetraploid (4×) (Fig. 1, Figs. S1 and S2). The branches of 2× continued to grow (Fig. 1a), and its axillary buds developed into new branches in the growing season (Fig. S1). In contrast to those of 2×, more than 80% of the axillary buds of 4× did not burst (Fig. S1e) or did not grow into a complete branch due to tip abscission (Fig. S1F–H). Surprisingly, the terminal buds of 4× also ceased growth, then changed from green to yellow and gradually became brown and abscised (Figs. 1c and S2). The 4× plants were propagated from at least five lines and showed the same morphology. However, this phenotype was not observed in the other grapevines.

Anatomical structure analysis revealed that the stem comprised four parts (Fig. 1d–f). The epidermis contained a single layer of small-round cells constituting the outermost layer of the stem. Under the small-round cells were round cortex cells, which were larger than epidermis cells but smaller than pith cells. The vascular bundles contained shuttle-shaped phloem cells in the outer region, and the inner xylem cells were arranged in a ring. The space in the cortex and around the vascular bundles was filled with large pith cells. The vascular bundles extended along the epidermis of the stem and petiole to the terminal buds and leaves (Fig. 1d, e). In the 4× grapevine, the AZ appeared at the base of the terminal bud and led to the cessation of the growth of the terminal bud as a result of the vascular bundles extending to the terminal bud being interrupted (Fig. 1f).

### Differentially expressed genes between 2× and 4×

The gene expression values in 2× and 4× were significantly highly correlated (Fig. S3A, *R*^2^ = 0.969, *P*_fdr_ < 2.2e^−16^). Comparison of the gene expression values between 2× and 4× revealed that 572 genes showed significant decreases, while 594 genes showed significant increases (*n* = 3, *P*_fdr_ < 0.05, Fig. S3B). In addition, 17,371 genes were not differentially expressed between the 2× and 4× grapevine terminal buds (non-diff genes), and 11,434 were not expressed in the buds (FPKM < 1, Fig. S3B).

**Fig. 2:**
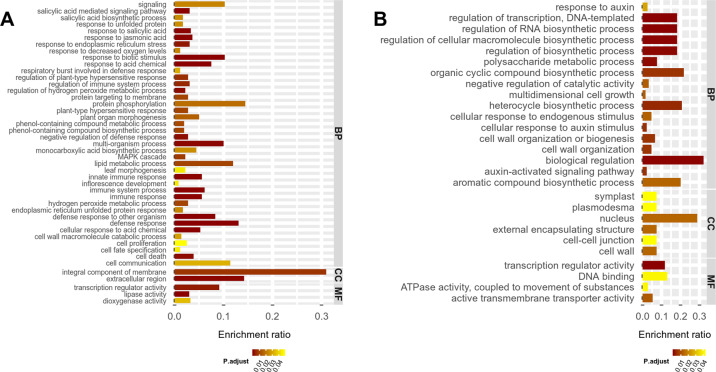
GO enrichment for differentially expressed genes. The significance and enrichment ratio for GO terms involved in 4× increased (**a**) and decreased (**b**) genes

The genes showing increases in 4× were enriched in 49 significant Gene Ontology (GO) terms, whereas the genes showing decreases in 4× were enriched in 33 significant GO terms (*P*_fdr_ < 0.05) (Fig. 2a and b, Table S4). As shown in Table S4, the greatest proportion of genes showing increases in 4× were involved in biological processes, including the response to biotic stresses (e.g., drought) and abiotic stresses (e.g., fungal attacks), and were more significantly enriched (Fig. 2a). The dominant GO terms among the genes showing decreases in 4× were biological metabolism and biosynthesis. Some sub-GO terms were correlated with the regulation of transcription, gene expression, and RNA metabolism. Others were correlated with the regulation of nitrogen compound metabolism, macromolecule biosynthesis, and cellular metabolism (Fig. 2b).

### Single-base resolution maps of DNA methylation for 2× and 4×

To examine the potential role of DNA methylation in WGDs, we performed WGBS for both the 2× and 4× grapevine tips. This process generated 72.9 and 67 million paired-end reads for 2× and 4×, respectively. The reads were then mapped to the *V. vinifera* genome. The average depths at cytosine sites were 12.86× and 11.88× in 2× and 4×, respectively. The 2× and 4× grapevines showed similar read coverage distributions in each context (Fig. S4D). The detected cytosine rates for 2× and 4× were 67.18% and 66.45%, respectively (Table S5).

We identified 22.34 and 21.11 million methylcytosines (mCs) from the total WGBS reads in the 2× and 4× grapevines, respectively (Fig. S4A). The largest proportion of mCs were in CHH context (13.67 and 12.75 million for 2× and 4×, respectively, Fig. S4B). The methylation rates of 2× and 4× in the CHH context were 16.5% and 15.57%, respectively (Fig. S4C). The second highest proportion of mCs were in CG context (4.45 and 4.3 million for 2× and 4×, Fig. S4B), which presented the highest methylation rates (34.21% and 33.42% in 2× and 4×, respectively, Fig. S4C). The numbers of mCs in the CHG context were 4.22 and 4.05 million in 2× and 4×, respectively (Fig. S4B), and the corresponding methylation percentages were higher than 30 (Fig. S4C).

### The autotetraploid grapevine methylome was conserved relative to that of its diploid parent

mC densities were calculated against 50 kb tiled bins in the genome. The 2× and 4× grapevines presented similar mC density and average methylation level distributions. The intergenic regions showed higher methylation densities and average methylation levels compared with the gene regions (Fig. S5).

The gC densities ranged from 0.1 to 0.5, whereas the total mC densities ranged from 0 to 0.14. Although the mC densities in each context showed a similar range, the counts of bins that presented higher mC densities were greater in CHH than in CHG and CG contexts. Additionally, a large proportion of bins presented low mC densities in CG and CHG (Fig. S6). The total mC densities were significantly correlated in 2× and 4×, and they were also significantly correlated in each context. The mC densities were not correlated with the gC densities (Fig. S7).

The average methylation levels in different contexts showed similar distributions between 2× and 4× (Figs. S5B and S7). The average levels of total mC for both 2× and 4× reached 7% (Fig. S8). mCs in the CG context presented the highest average methylation levels, reaching 3.5% (Fig. S8), followed by those in the CHG context, reaching 3%. Most of the average methylation level values were below 1% (Fig. S8), and mCs in the CHH context showed the lowest average methylation levels, of less than 1.5% (Fig. S8). The average mC levels were significantly correlated between 2× and 4× (*R*^*2*^ > 0.99, *P* < 2.2e-16) (Figs. S5B and S9).

### Methylome variation between ‘Jingyanjing’ and its autotetraploid

**Fig. 3:**
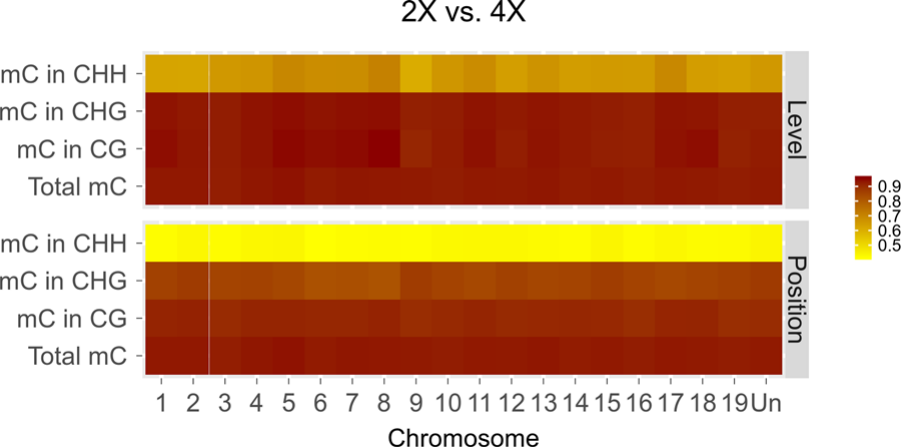
Heatmap of the correlation coefficient of mC levels and mC positions between diploid and autotetraploid. The *x*-axis refers to each chromosome, and the *y*-axis refers to total mC and mC in CG, CHG, and CHH, respectively

The total mCs of 2× and 4× were highly correlated in terms of both methylation levels and mC positions (*R*^*2*^ > 0.9) (Fig. 3). The mCs in 2× and 4× were also highly correlated in CG and CHG contexts, and the correlation coefficients were higher than 0.9 for CG (Fig. 3) and between 0.85 and 0.9 for CHG (Fig. 3). However, mCs in the CHH context were not correlated (Fig. 3).

The methylome showed a large variation between 2× and 4×, with a variance of 46.87 (Fig. S10). The variance in the mCs exhibiting an increased methylation level (24.6%) was larger than that in the mCs exhibiting a decreased methylation level (22.3%) (Fig. S10). The mCs showed the greatest variation in the CHH context, and the variances in the mCs presenting increased and decreased methylation levels in this context were 10% and 8.89%, respectively. The variance of the mCs showing both increasing and decreasing methylation levels in the CHH context with a change of more than twofold exceeded 2% (Fig. S10). The second highest variation was observed for the mCs in the CHG context, where the variances of the mCs exhibiting increasing and decreasing methylation levels in the CHG context were 7.42% and 6.87%, respectively. The variances in the mCs showing increased and decreased methylation levels in the CHG context with a change of more than twofold were all ~1% (Fig. S10). Our results showed that the variation of mCs in the CG context was the smallest. The variances of the mCs showing increased and decreased methylation levels in this context were 7.1% and 6.5%, respectively. However, there only 0.36% of increased mCs and 0.3% of decreased mCs in the CG context showed a change of more than twofold (Fig. S10).

### Distribution of DMRs

We identified 1.14 × 10^5^ DMRs, 41 and 6 of which were in CG and CHG contexts, respectively (Table S6). This result indicated the high conservation of whole-genome methylation in CG and CHG contexts during WGD. The DMRs showing increased and decreased methylation levels presented similar length distributions, and more than 80% of the DMRs were less than 200 bp (Fig. S11). The count percentage of DMRs that were located in gene regions were 55.3. The DMRs with increased and decreased methylation levels also showed similar location distributions in gene regions (Fig. S12). The percentages of DMR-containing genes were 67.51, 66.96, 70.65, and 56.19 for the 4×-increased, 4×-decreased, non-differentially expressed and non-expressed genes, respectively.

### Gene body hypo-DMRs increased gene expression in 4X

**Fig. 4:**
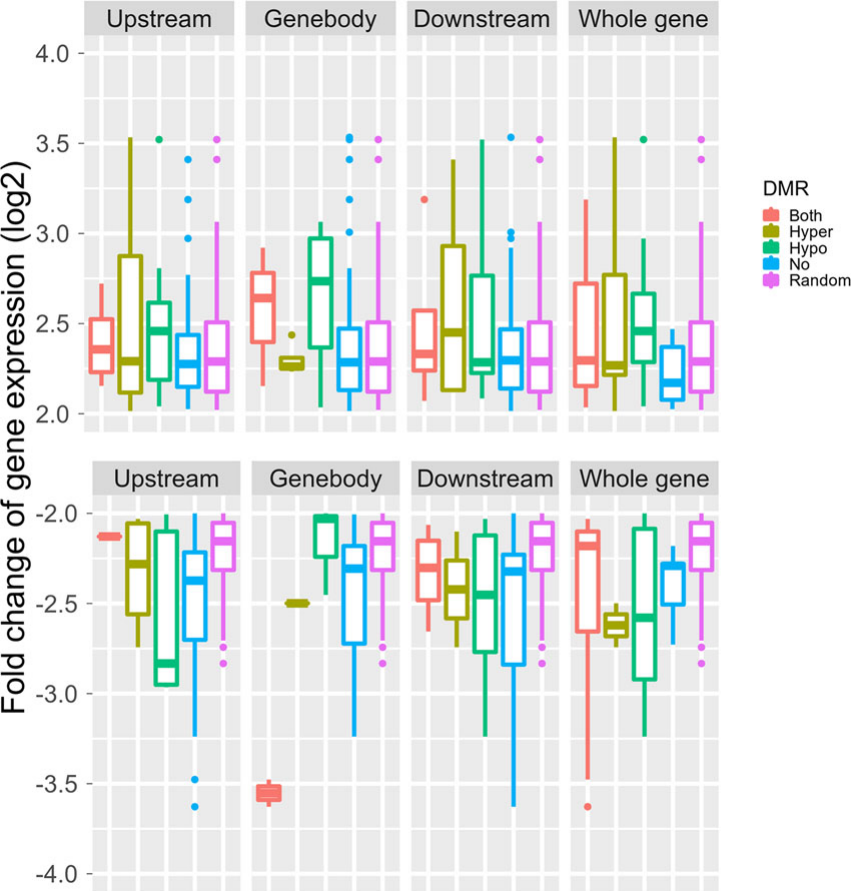
Relationship between DMRs and fold change of genes. Boxplot depicting log2-fold change in differentially expressed genes (≥4-fold change) and their DMRs in each and whole gene region. A group of randomly selected genes is also shown (random)

All the genes were divided into four groups according the types of their DMRs (hyper, hypo-, both hyper- and hypo-DMRs, and no-DMR). These four groups of genes showed similar expression patterns (Fig. S13). Figure 4 shows the relationship between the fold changes of the DEGs (≥4-fold change) and their DMRs. Among the genes showing increases in 4×, hypo-DMRs in the gene body were associated with a higher gene expression fold change than was observed in the hyper-DMR, no-DMR, and randomly selected genes (Fig. 4). Among the genes showing decreases in 4×, hyper-DMRs in the gene body were associated with a higher gene expression fold change than was observed in the hypo-DMRs, no-DMR, and randomly selected genes (Fig. 4). In the upstream and downstream regions and whole genes, the four groups of genes showed no differences in gene expression fold changes (Fig. 4).

**Fig. 5:**
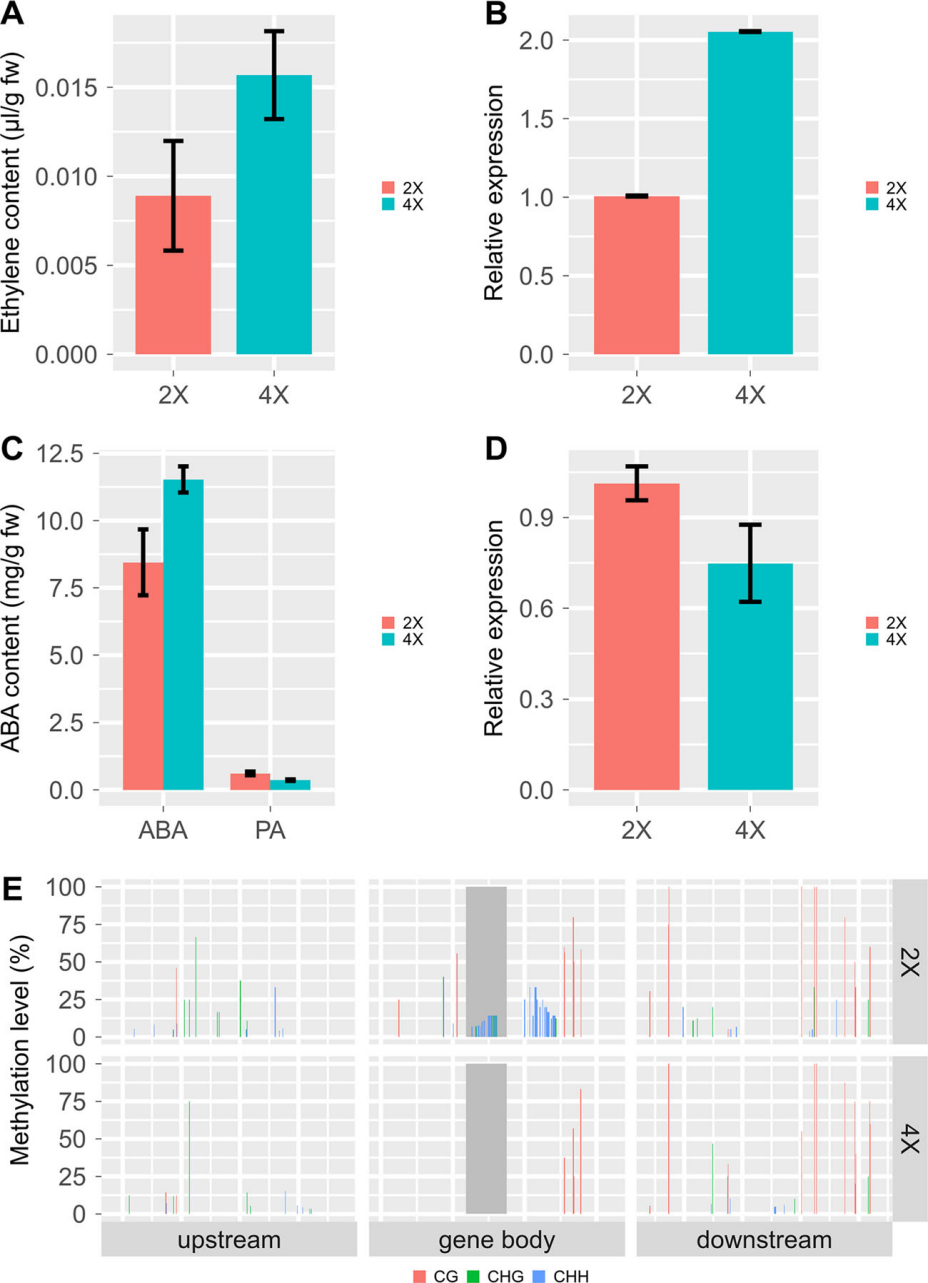
Plant hormone contents and their correlated genes. **a** Ethylene contents in ‘Jingyanjing’ (2×) and its autotetraploid (4×). The 4× grapevine released significantly more ethylene than 2× (*P* < 0.05). **b** The *ACO1* gene was more significantly expressed in 4× than in 2× (*P* < 0.05). **c** The contents of ABA were significantly higher in 4× than in 2×, and the contents of PA were significantly lower in 4× than in 2× (*P* < 0.05). **d** *CYP707A* was significantly less expressed in 4× than in 2× (*P* < 0.05). **e** Methylation status of *ACO1* in 2× and 4×. A DMR, which is shown as a bar in dark gray, is found in its gene body. Error bars indicate SE from three individual replicates

The expression levels of twelve genes containing hypo- and/or hyper-DMRs in their gene body were increased or decreased more than fourfold in 4× (Table S7). Among the three genes showing decreases in 4×, two contained both hypo- and hyper-DMRs, and one contained only a hyper-DMR. Additionally, six of the nine genes showing increases in 4× contained only hypo-DMRs, and the other three contained both hypo- and hyper-DMRs (Table S7). Surprisingly, an ACC oxidase (*ACO1*) was included in these genes showing increases in 4× (Fig. 5e).

The DMR in the *ACO1* gene body between 2× and 4× was verified by BS-PCR in the top tips of other diploid, autotetraploid, and allotetraploid grapevines. There was no significant difference between the other diploid and autotetraploid varieties. This region showed higher methylation in the allotetraploid grapevine top tips (Fig. S14).

### Variation of plant hormones and corresponding genes between 2× and 4×

**Fig. 6:**
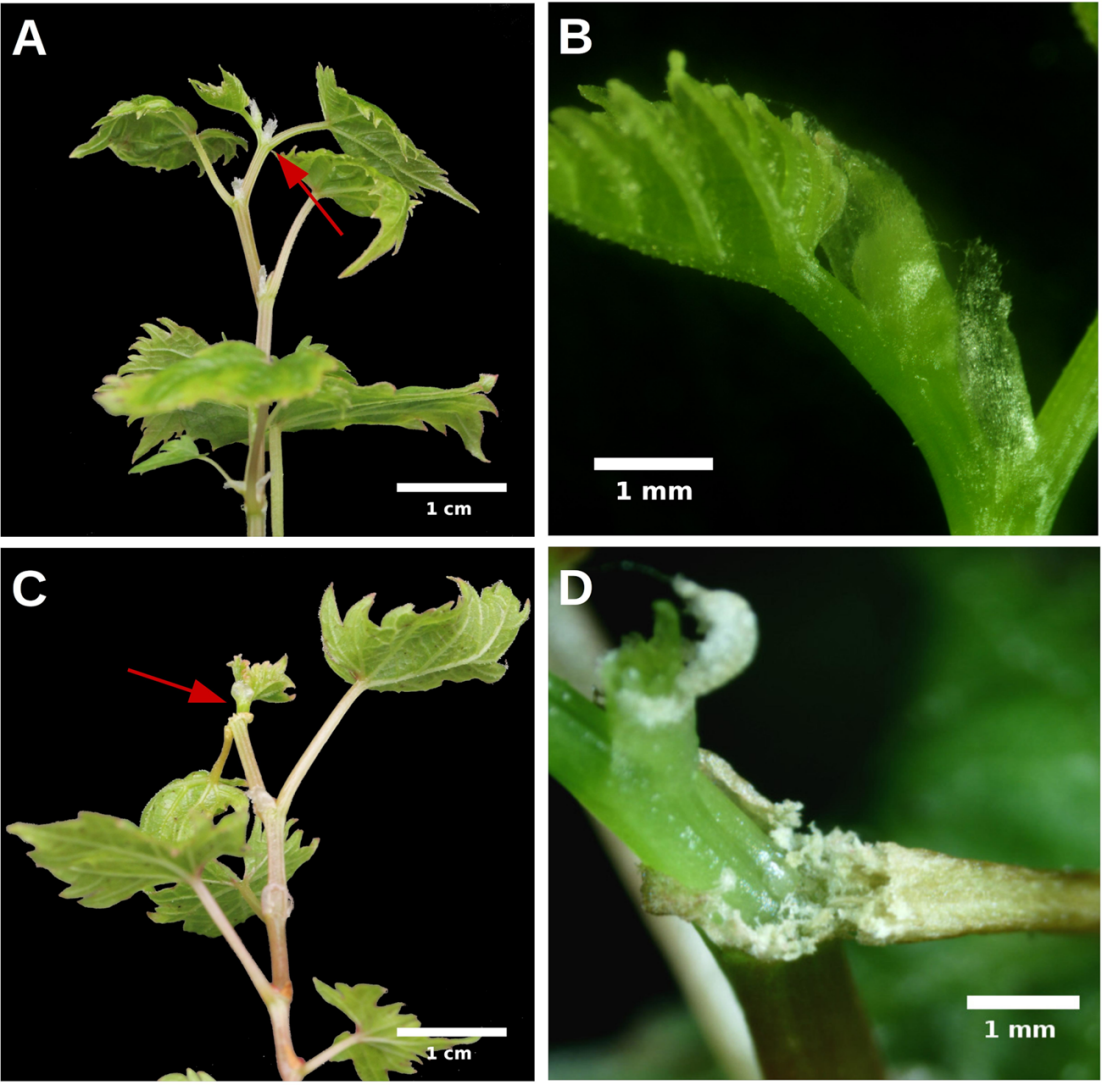
The ‘Jingyanjing’ grapevine (2×) and its autotetraploid (4×) respond to 50 µl/L ethylene. **a**, **c** 2× and 4× after treatment with ethylene. **b**, **d** 10× magnification for the red arrows pointed part of (**a**) and (**c**). The abscission zone clearly appeared in 4× (**d**)

**Fig. 7:**
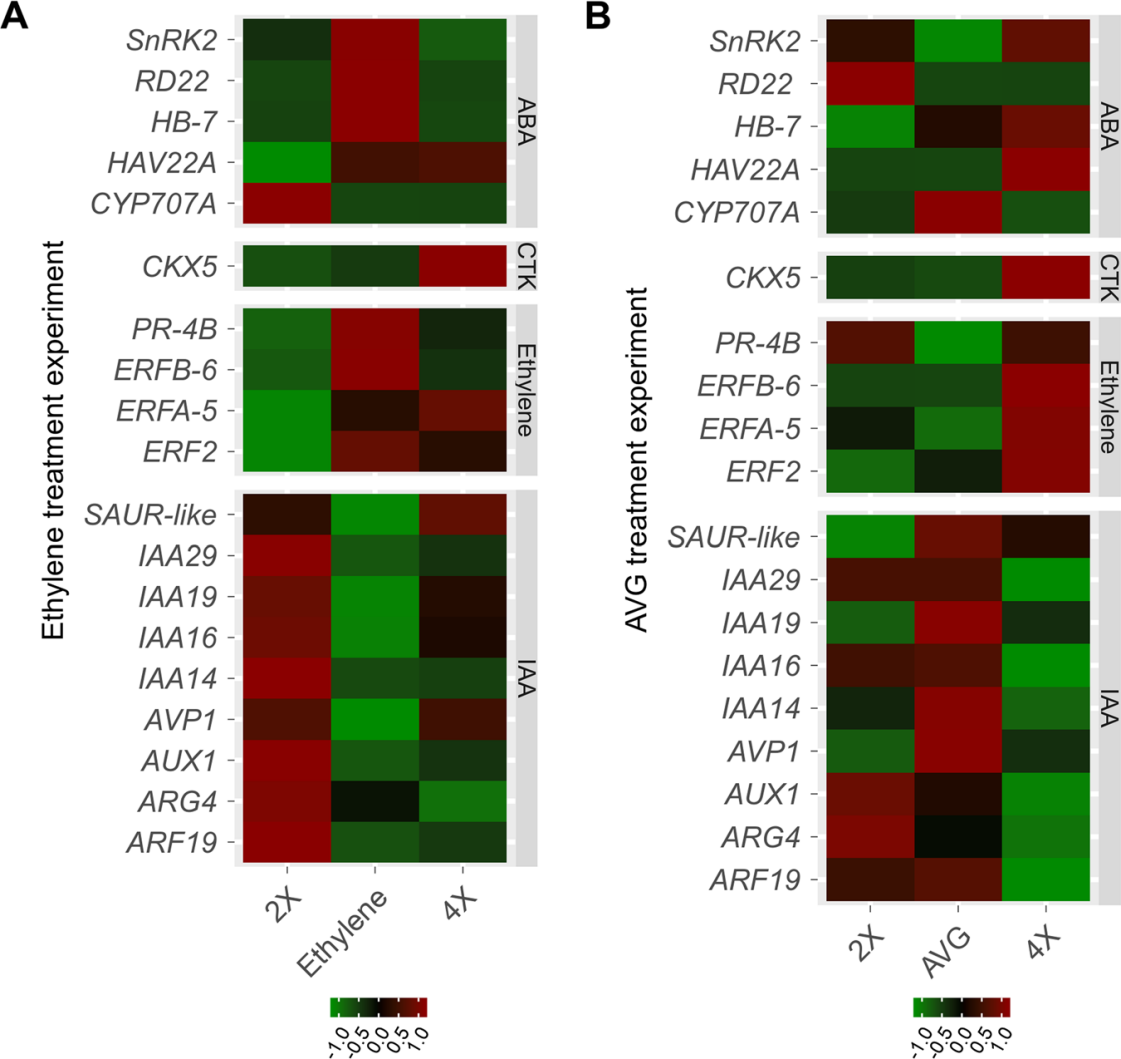
Expression patterns of ethylene response-correlated genes in ethylene and AVG treatment experiments. **a** Expression patterns of ethylene response-correlated genes in ‘Jingyanjing’ (2×), 2× treated with ethylene (ethylene), and its autotetraploid (4×). These genes showed similar expression patterns in 4× and ethylene-treated 2× compared with those in 2×. **b** Expression patterns of ethylene response-correlated genes in 2× and 4× treated with AVG (AVG) and 4×. These genes in 2× and AVG-treated 4× expressed similar expression patterns compared with those in 4×

The ethylene contents were significantly different between 2× and 4×. The 2× grapevine released 0.009 µl/g ethylene in 24 h, whereas 4× released 0.016 µL/g ethylene over the same time period (*n* = 3, *P* < 0.05) (Fig. 5a). The *ACO1* presented a similar expression tendency to ethylene (*n* = 3, *P* < 0.05) (Fig. 5B). In addition, the expression levels of 10 ethylene response genes were increased in 4X, and four of these genes were *EREBP* members containing AP2 domains belonging to the ethylene response factor (*ERF*) family. Genes related to ROS resistance (*CAT2-1*) and pathogen-related 4 (*PR-4* and *PR-4B*), GDSL lipase (*GLIP1* and *GLIP2*) and basic chitinase (*HCHIB*) genes, which have been reported to be responsive to ethylene^29–32^, showed increases (*n* = 3, *P* < 0.05) (Fig. S15). In tissue culture plantlets, the expression levels of three *ERF* genes and *PR-4B* were increased in 4×. The 4× grapevine was more sensitive to exogenous ethylene than 2×. After treatment with ethylene, AZs were present in 4× in the flakes of the terminal buds and at the base of the top leaf alongside the terminal, which were not found in 2× (n = 3) (Fig. 6). The expression levels of these three *ERF* genes and *PR-4B* were increased after treatment with ethylene in 2× (*n* = 3, *P* < 0.05) (Fig. 7a), whereas they were significantly reduced by the ethylene inhibitor aminoethoxyvinylglycine (AVG) in tissue culture plantlets of 4× (*n* = 3, *P* < 0.05) (Fig. 7b).

The measured ABA and PA contents are shown in Fig. 5c. The 4× grapevine contained significantly more ABA than the 2× grapevine, and the PA content in 4× was significantly less than that of 2× (*n* = 3, *P* < 0.05). The expression level of the ABA degradation gene *CYP707A* was increased by ethylene^33^. In this study, it was found to be significantly decreased in 4X (*n* = 3, *P* < 0.05) (Fig. 5d). Seven ABA response genes and *SnRK2* showed increases in 4× (*n* = 3, *P* < 0.05) (Fig. S14). In the 4× tissue culture plantlets, four of these seven genes showed increases, while *CYP707A* was decreased (*n* = 3, *P* < 0.05) (Fig. 7). The four ABA response genes showed increases, whereas *CYP707A* was decreased after ethylene treatment in 2×; in contrast, these ABA response genes showed decreases, and *CYP707A* was increased in 4× after AVG treatment (*n* = 3, *P* < 0.05) (Fig. 7).

Three auxin transporters (*AUX1*, *AVP1*, and *PGP1*) and nine auxin response genes showed decreases in 4× (*n* = 3, *P* < 0.05) (Fig. S15). These genes were enriched in a significant GO term (auxin-mediated signaling pathway, Fig. 2b). Among the nine auxin response genes, six belonged to the *Aux/IAA* gene family, *KNAP2* is a homeobox gene, and the two others are *SAUR-like* genes. In tissue culture plantlets, nine of the 12 genes showed decreases in 4×. The *PGP1* transporter and a *SAUR-like* gene were not significantly different between 2× and 4×. These nine genes showed decreases in 2X after treatment with ethylene, and they showed increases under AVG treatment in 4× (*n* = 3, *P* < 0.05) (Fig. 7).

A cytokinin oxidase gene (*CKX5*) presented significantly increased expression in 4×, and four corresponding cytokinin genes, including two *CYCD3* genes, a *HAT22* gene, and a *CRF4* gene, showed decreases in 4× (*n* = 3, *P* < 0.05) (Fig. 7, Fig. S15). CKX activity is reported to be increased by ethylene^34^. In our study, we found that ethylene treatment increased the expression of *CKX5* in 2×, whereas AVG decreased its expression in 4× (Fig. 7). CK at >2 mg/L induced lateral shoot growth in 4× and increased lateral shoot growth in 2× (*n* = 3, *P* < 0.05) (Fig. S16).

## Discussion

### DNA methylation conservation during grape genome duplication

DNA methylation on cytosines plays a critical role in epigenetics, and mCs are highly conserved over generations. For example, nitrogen deficiency-induced epigenetic modifications in rice are inheritable^35^ and Verhoveven et al. revealed that plant hormones and salt stress-induced methylation variation are faithfully transmitted to asexual offspring in dandelion^36^. A recent study demonstrated that the methylome of *Arabidopsis* is stable under drought over five generations^37^. A study in *Cymbopogon* including autotetraploids revealed that methylcytosines were increased during genome duplication^38^. However, in that study, the authors used an immunodetection method that reflects the total amount of methylcytosines, while the methylation level of each cytosine remains unknown. Some studies have reported that mC sites are comparable between diploids and autotetraploids^39,40^. However, these studies used only a subset of methylation-sensitive amplified polymorphism markers, which cannot reflect the genome-wide methylation and methylation variation in CG, CHG, and CHH contexts. In this study, WGBS was employed to produce single-base resolution DNA methylation maps for ‘Jingyanjing’ and its autotetraploid. The results showed that DNA methylation was highly conserved at the 50 kb level during polyploidization in grapes (Fig. S5). At the single-base level, the positions and levels of total mC, mC in CH, and mC in CHG were highly conserved (Fig. 3). More than 0.1 million 100 bp DMRs were identified in the CHH context, and trace DMRs were found in CG and CHG contexts. These results also indicated the conservation of the methylome in CG and CHG contexts at the 100 bp level. Plants have an accurate system for maintaining a stable methylation status transgenerationally^41^. Our results revealed that the system maintaining DNA methylation also ensures DNA methylation stability in CG and CHG contexts.

DNA methylation variations have been observed during genome polyploidization in strawberry and *Brassica rapa* by using MSAP^42,43^. A previous study employed WGBS to detect epigenetic variance between a diploid and an autotetraploid produced from it in rice and identified 100 bp DMRs, a few of which are in CG and CHG contexts^44^. In the present study, epigenetic changes were also observed by using WGBS at the single-base level. In contrast to previous works, the present study revealed mC variance in the CHH context at both the single-base and 100 bp levels (Table S6). DNA methylation polymorphism has been detected within allotetraploid cotton (*Gossypium hirsutum*)^45^. We also revealed methylation variation in the *ACO1* gene body in diploid and autotetraploid and allotetraploid grapevines.

### Gene body methylation modification was negatively correlated with gene expression

DNA methylation changes during polyploidization^40,43,44^. However, the effects of DNA methylation variation on gene expression during polyploidization remain unclear. In the present study, DEGs and DMRs were identified between ‘Jingyanjing’ and autotetraploid grapevines produced from this accession, and the results revealed that DMRs were negatively correlated with gene expression during genome duplication in grape. DNA methylation in the promoter region of a gene can directly repress its expression by inhibiting transcription factor binding^39^ or may indirectly decrease its expression by inhibiting histone modifications^46^. DNA methylation modifications in promoters were detected between ‘Jingyanjing’ and its autotetraploid, but the four gene groups (hypo-, hyper-, both, and no-DMRs in the promoter) and randomly selected genes showed similar expression patterns (Fig. 4, Fig. S13). Previous studies established mathematical models and revealed a positive correlation between gene body methylation levels and expression values in human cell lines^27^. However, the effects of gene body methylation variation on gene expression are poorly understood. In this study, the DMRs located in gene body regions were negatively correlated with gene expression during genome duplication in grapevine.

### Relationship between DNA demethylation and self-pruning in autotetraploid grapevine

An *ACO1* gene containing a hypo-DMR in its gene body was upregulated in 4X, which released more ethylene than ‘Jingyanjing’. Ethylene inhibits auxin transportation and breaks down auxin signaling pathways. Increases in ABA activity during plant organ abscission are widely reported^22,24^. Our results indicated that increased release of ethylene might inhibit the expression of auxin transport and signaling pathways in the 4X grapevine. Increasing the release of ethylene might also reduce ABA degradation and increase ABA production.

Previous studies have found that AZ formation is induced by ethylene during self-pruning^18^. In the present study, we also observed AZs in autotetraploid grapevine tips. RNA-Seq and qPCR data showed that *ACO1* played an important role during self-pruning in the 4X grapevine. The ethylene and AVG treatment results revealed that the correlated ethylene response genes were up- and downregulated in ‘Jingyanjing’ and its autotetraploid, respectively. These results indicated that the upregulation of ethylene-responsive genes was due to the upregulation of *ACO1*, which increased the release of ethylene in 4X. After being treated with ethylene, AZs were present in 4× but not in 2X (Fig. 6). This might be due to the endogenous ethylene content being higher in 4×, as it presented a lower threshold in response to exogenous ethylene.

## Conclusion

Autotetraploid ‘Jingyanjing’ grapevine showed little and/or abnormal lateral shoot growth and self-pruning morphologies, which are potentially valuable agronomic traits in grape. During grapevine WGD, the methylome was conserved, and gene body methylation variation was negatively correlated with gene expression. The gene body demethylation of *ACO1* was correlated with self-pruning morphology in autotetraploid ‘Jingyanjing’.

## Materials and methods

Plant materials and treatments’Jingyanjing’ grapevine (2×) is the offspring of ‘Jingzaojing’ (*V. vinifera*) and ‘Jingxiu’ (*V. vinifera*). ‘Jingzaojing’ and ‘Jingxiu’ were planted in 1993. ‘Jingyanjing’ grapevine was generated during 1997. It was induced to form tissue culture plantlets, and the tissue culture plantlets were induced to form an autotetraploid^47^. In this previous study, the stems of tissue culture plantlets with axillary buds were immersed in 2000–4000 mg/L colchicine for 0.5–4 days. Then, the treated stems were inoculated onto B5 medium. After the axillary buds sprouted, their ploidy levels were detected by using flow cytometry. The optimum conditions for inducing autotetraploid grapevines are the treatment of the axillary buds with 3000 mg/L colchicine for three to four days in different varieties. The rates of induction range from 16.7 to 23.3%. Tissue-cultured 2× and 4× ‘Jingyanjing’ grapevines were planted in the experimental vineyard of the germplasm repository for grapes in the Institute of Botany of the Chinese Academy of Sciences in Beijing in 2006. All the vines underwent standard fertilization, irrigation, pruning, and pest and disease control procedures.

In our study, the plant materials used for the tissue culture of plantlets were prepared as previously described by Mukherjee et al. and propagated for two months. Woody Plant Media supplemented with 20 g/L sucrose, 6 g/L agar, and 0.05 mg/L IAA were used for the tissue culture of plantlets (Duchefa Biochemie, Haarlem, the Netherlands). The pH of the culture media was adjusted to 5.8 before autoclaving at 15 psi (121 °C) for 20 min. The cultures were maintained at 25 ± 2 °C with a 16 h/8 h day/night cycle with a light intensity of 50 µmol/(m^2^s).

To treat the plantlets with ethylene, plantlets that had been grown for four weeks were sealed in 1 L bell jars. Then, 50 µL of pure ethylene was injected into the jars. The controls were sealed in jars without ethylene (*n* = 3). After 48 h of treatment, the terminal buds of the plantlets were collected to detect gene expression. They were also observed under a microscope to detect AZ formation. The plantlets were further examined after treatment with the ethylene biosynthesis inhibitor AVG (Sigma Ltd., UK). Four-week-old plantlets were treated with 5 µmol/L AVG for 48 h (*n* = 3). Then, they were collected to detect gene expression by qPCR. To investigate the effects of cytokinin on the growth of the vines, we added 4 mg/L autoclaved 6-benzyl amino purine (BAP) to plantlets that had been grown for (*n* = 3). After 4 weeks, the BAP-treated 2× and 4× plantlets, as well as their untreated controls, were observed and compared.

### Anatomical observation

For anatomical observation, stem tip samples of 2×, growing 4×, and the autotetraploid whose growth had ceased were collected and fixed in FAA (5% formaldehyde, 5% acetic acid, and 63% ethanol (v/v/v)) overnight at room temperature. During this period of development, the terminal buds of the shoots were 7–10 mm in length; the diameter of the bud was 7–10 mm; and the diameter of the stem tip was 2–4 mm; and the buds was packed within a folded leaflet. The samples were dehydrated in a graded ethanol series of ethanol solutions, infiltrated with xylene, and then embedded in paraffin wax^48^. Sections were cut to a thickness of 10 μm by using a Leica RM2235 rotary microtome (Leica Instruments Company Ltd., Shanghai, China) and then stained with safranin for 8 h and toluidine blue for 30 s. The slides were mounted with synthetic resin, and the sections were observed and photographed under an Olympus BX41 microscope (Olympus Optical Company Ltd., Tokyo, Japan).

### Plant hormone and metabolite quantification

Tissue culture plantlets that had been grown for 4 weeks were sealed in 400 mL tissue culture bottles. After 24 h of cultivation, 1 mL of gas from each bottle was analyzed with an SP2100A gas chromatograph (Beijing Beifen Ruili Analytical Instrument Co. Ltd, Beijing, China) equipped with an HP-5 capillary column (30 m × 0.53 mm × 40 μm) and a flame ionization detector, which were controlled at 100 °C and 50 °C, respectively. Then, the ethylene concentrations were normalized by the total plantlet weight in the bottle (*n* = 3).

The samples for the analysis of ABA and its metabolites were collected one week before autotetraploid terminal bud abscission. The length and diameter of the buds were 7–10 mm. We performed three biological replicates for the diploid and autotetraploid plants. The terminal buds from three independent vines were mixed together as a biological replicate. ABA and its metabolites were quantified as described by Speirs et al.^49^. The terminal buds of both 4X and 2X were freeze-dried and ground to a fine powder. A 50–100 mg sample was used for extraction, to which an internal standard containing deuterated PA and ABA was added. Phenomenex SPE columns (8B-S100-UAK) were used to perform a “clean-up” step prior to the analysis. The samples were loaded onto the columns, washed with 20% aqueous methanol, and then eluted with 90% aqueous methanol. An aliquot of the eluate was dried in a vacuum centrifuge.

The ABA concentration was quantified by liquid chromatography-mass spectrometry (LC-MS/MS). The dried extracts were dissolved in aqueous acetonitrile (10% with 0.05% acetic acid) and analyzed by LC-MS/MS (Agilent 6410). Separation was carried out in a Phenomenex C18 column at 40 °C using a gradient from 10% acetonitrile and 90% water to 90% acetonitrile and 10% water. Compounds were identified by their retention times and through multiple reaction monitoring.

### RNA isolation, qPCR, and RNA-Seq library construction and sequencing

The same samples used for ABA analysis were used for RNA-Seq (*n* = 3). We isolated total RNA from ground terminal bud samples (100 mg per sample) using a Sigma RNA Mini-Prep kit following the manufacturer’s instructions (Zymo Research Corporation, Irvine, CA, USA). The extracted RNA was treated with DNase (Applied Biosystems/Ambion, Austin, TX, USA). Pure RNA was used for qPCR and RNA-Seq. The quality and concentration of the RNA were measured with a Nanodrop ND-1000 Spectrophotometer (Thermo Fisher Scientific Inc., USA).

For cDNA synthesis, 1 μg of RNA was reverse transcribed using SuperScript® III Reverse Transcriptase (Invitrogen) in accordance with the manufacturer’s instructions. For the qPCR assay, the cDNA was diluted 1:10 with ddH_2_O. Gene-specific primers were designed according to the reference CDS using PerlPrimer (Marshall, 2004). β-actin was used as the reference gene. The reactions were carried out by using a StepOnePlus real-time PCR instrument (Applied Biosystems). Primer specificities were tested via qPCR melting curve analysis. The qPCR mixture contained 10 μL of 2× SYBR Green I Master Mix (Vazyme), 0.2 μL of each diluted primer (10 μM), 2.6 μL of ddH_2_O, and 7 μL of diluted cDNA template. The transcription levels of each gene were normalized against the average for β-actin. We performed three biological replicates for each sample and three technological replicates for each biological replicate. The primers used in this study are listed in Table S1.

The terminal bud samples were collected and sent to 1GENE (Hangzhou, China) for RNA extraction, RNA-Seq library construction and sequencing. There were three replicates for both 2× and 4×, and each replicate was pooled from at least three terminal buds.

### Whole-genome bisulfite sequencing and BS-PCR

The samples for WGBS were collected one week before 4× terminal bud abscission. The 2× and 4× samples were pooled from the terminal buds of three diploid and three autotetraploid vines, respectively. Then, these two samples were ground to a powder, and total DNA was extracted by using a Plant Genome DNA Purification Kit (TIANGEN Biotech, Beijing, China) and sent to Novogene (Beijing, China) for bisulfite treatment, library preparation, and next-generation sequencing on the Illumina HiSeq 4000 platform (Illumina HiSeq. 4000). To identify the DMR in the *ACO1* gene body in ‘Jingyanjing’ grapevines and further examine whether this DMR appeared between other diploid and autotetraploid varieties, bisulfite sequencing PCR (BS-PCR) analysis was performed for 11 other diploid, autotetraploid, and allotetraploid accessions to verify the DMR in the *ACO1* gene body (Table S2). Genomic DNA was isolated from the terminal bud samples of these grapevines with the Plant Genome DNA Purification Kit (TIANGEN Biotech, Beijing, China). Then, the DNA was modified with the EZ DNA Methylation-Gold Kit (Zymo Research Corporation, Irvine, CA, USA). The BS-PCR primers were designed to target the 100 bp sequence flanking this region with PerlPrimer (Marshall, 2004). PCR was performed by using Zymo Taq Premix (Zymo Research Corporation, Irvine, CA, USA). The PCR products were purified using the V-ELUTE Gel Mini Purification Kit (Beijing Zoman Biotechnology, Beijing, China). Then, the purified products were cloned into the pLB vector (TIANGEN Biotech, Beijing, China). We performed three replicates for each sample. Each replicate was pooled from at least three terminal buds. For each sample, at least 10 clones were sequenced using the Sanger method to analyze the methylation status.

### RNA-Seq data analysis

The reads were trimmed using FASTX-Toolkit (http://hannonlab.cshl.edu/fastx_toolkit/). The nucleotides at the beginning and end of each read whose quality thresholds were lower than 13 were trimmed. Only the clean reads were used for subsequent analysis. In silico expression profiles were generated using TopHat (version 2.0.14, http://ccb.jhu.edu/software/tophat), which is an automated pipeline that uses Bowtie2 (http://bowtie-bio.sourceforge.net) to align the reads of each library against the genome sequence of *V. vinifera* on the basis of its annotation (http://plants.ensembl.org/Vitis_vinifera/Info/Index). The mapping information is listed in Table S3. Then, DEGs between 2× and 4× were identified using Cufflinks (version 2.2.1). Genes with an FDR < 0.05 were regarded as differentially expressed genes (DEGs).

### Methylation data analysis

After removing low-quality reads (Q score < 28), 150 nt paired-end WGBS reads were mapped to the *V. vinifera* genome by using Bimark software. Only cytosines from both 2× and 4× that were covered by at least three reads were considered and counted. The methylation level of individual cytosines was calculated as the ratio of mC to the total cytosines [mC/(mC+un-mC)]. The fold change at each site was calculated by comparing the methylation ratio of 4× to the methylation ratio of 2×. An mC fold change greater than 1.2 was considered to indicate differential methylation. The densities and average methylation levels were calculated in 50 kb units.

### Identification of DMRs

We tiled the whole genome into 100 bp bins with 25 bp sliding windows. The Kruskal-Willis rank sum test^50^ was performed on each bin for the mCs in CG, CHG, and CHH contexts to identify significant differences between 2× and 4×. The *P-*value of the bins was adjusted by using BH multiple test correction. Bins with a *P*_fdr_ value of less than 0.05 were defined as DMRs. The ratio of the average methylation levels of a DMR between 4× and 2× was defined as the fold change of the DMR. Adjacent DMRs showing the same trend were collapsed into a single DMR. Then, the DMRs were annotated in the upstream 2 kb, downstream 2 kb, and gene body regions.

## Supplementary information

Figure S1

Figure S2

Figure S3

Figure S4

Figure S5

Figure S6

Figure S7

Figure S8

Figure S9

Figure S10

Figure S11

Figure S12

Figure S13

Figure S14

Figure S15

Figure S16

Table S1

Table S2

Table S3

Table S4

Table S5

Table S6

Table S7

## Data Availability

All RNA-seq and WGBS data from this study are available from the NCBI Gene Expression Omnibus (GEO) under the accession numbers GSE119442 and GSE119632, respectively. The grapevines used in our study are common *Vitis* species. They are not classified as endangered species. They are conserved in the experimental vineyard of the germplasm repository for grapes in the Institute of Botany of the Chinese Academy of Sciences in Beijing. The grapevines used in this study are publicly available for non-commercial purposes.
